# Tri-μ-sulfato-κ^6^ *O*:*O*′-bis­[aqua­(1,10-phenanthroline-κ^2^ *N*,*N*′)indium(III)] dihydrate

**DOI:** 10.1107/S1600536810036330

**Published:** 2010-09-15

**Authors:** Fwu Ming Shen, Shie Fu Lush

**Affiliations:** a Department of Biotechnology, Yuanpei University, HsinChu 30015, Taiwan; b Department of General Education Center, Yuanpei University, No. 306, Yuanpei St, HsinChu 30015, Taiwan

## Abstract

In the title dinuclear In^III^ compound, [In_2_(SO_4_)_3_(C_12_H_8_N_2_)_2_(H_2_O)_2_]·2H_2_O, each In^III^ cation is coordinated by a 1,10-phenanthroline (phen) ligand, a water mol­ecule and three sulfate O atoms in a distorted InN_2_O_4_ octa­hedral geometry. Three sulfate anions bridge two In^III^ cations, forming the dinuclear entities. O—H⋯O and weak C—H⋯O hydrogen bonding is observed in the crystal structure. The crystal structure is further consolidated by π–π stacking between nearly parallel phen ring systems [dihedral angle = 4.2 (4)°], the centroid–centroid distance between benzene rings of adjacent phen ligands being 3.528 (9) Å.

## Related literature

For structures of indium complexes with Lewis base ligands, see: Ahmadi *et al.* (2008 ▶); Kalateh *et al.* (2008 ▶); Xiao & Zhan (2010 ▶). For the crystal structure of an indium compound with a sulfonate bridging ligand, see: Ramezanipour *et al.* (2005 ▶).
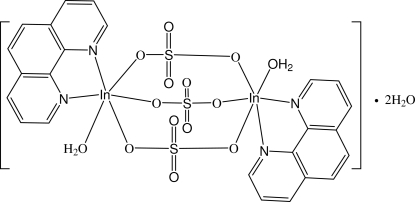


## Experimental

### 

#### Crystal data

[In_2_(SO_4_)_3_(C_12_H_8_N_2_)_2_(H_2_O)_2_]·2H_2_O
*M* *_r_* = 950.32
Triclinic, 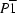

*a* = 10.5909 (2) Å
*b* = 12.0354 (2) Å
*c* = 13.3593 (3) Å
α = 79.904 (1)°
β = 79.606 (1)°
γ = 64.727 (1)°
*V* = 1505.32 (5) Å^3^
*Z* = 2
Mo *K*α radiation
μ = 1.83 mm^−1^
*T* = 295 K
0.18 × 0.12 × 0.04 mm

#### Data collection

Bruker SMART CCD area-detector diffractometer
Absorption correction: multi-scan (*SADABS*; Bruker, 2000 ▶) *T*_min_ = 0.789, *T*_max_ = 0.850
14402 measured reflections
5277 independent reflections
4377 reflections with *I* > 2σ(*I*)
*R*_int_ = 0.079

#### Refinement

*R*[*F* ^2^ > 2σ(*F* ^2^)] = 0.082
*wR*(*F* ^2^) = 0.224
*S* = 1.17
5277 reflections
406 parameters
H-atom parameters constrained
Δρ_max_ = 3.41 e Å^−3^
Δρ_min_ = −1.32 e Å^−3^

### 

Data collection: *SMART* (Bruker, 2000 ▶); cell refinement: *SAINT* (Bruker, 1999 ▶); data reduction: *SAINT*; program(s) used to solve structure: *SHELXS97* (Sheldrick, 2008 ▶); program(s) used to refine structure: *SHELXL97* (Sheldrick, 2008 ▶); molecular graphics: *ORTEP-3* (Farrugia, 1997 ▶); software used to prepare material for publication: *PLATON* (Spek, 2009 ▶).

## Supplementary Material

Crystal structure: contains datablocks global, I. DOI: 10.1107/S1600536810036330/xu5026sup1.cif

Structure factors: contains datablocks I. DOI: 10.1107/S1600536810036330/xu5026Isup2.hkl

Additional supplementary materials:  crystallographic information; 3D view; checkCIF report

## Figures and Tables

**Table 1:** Selected bond lengths (Å)

In1—N1	2.240 (11)
In1—N2	2.228 (11)
In1—O1	2.109 (9)
In1—O5	2.187 (9)
In1—O9	2.075 (10)
In1—O13	2.206 (9)
In2—N3	2.247 (12)
In2—N4	2.246 (11)
In2—O2	2.109 (9)
In2—O6	2.103 (10)
In2—O10	2.146 (9)
In2—O14	2.159 (9)

**Table 2:** Hydrogen-bond geometry (Å, °)

*D*—H⋯*A*	*D*—H	H⋯*A*	*D*⋯*A*	*D*—H⋯*A*
O13—H13*A*⋯O4^i^	0.81	1.94	2.602 (15)	138
O13—H13*B*⋯O12^ii^	0.84	1.94	2.637 (16)	139
O14—H14*A*⋯O8^iii^	0.82	1.85	2.667 (17)	173
O14—H14*B*⋯O16	0.87	1.76	2.565 (19)	152
O15—H15*A*⋯O12	0.90	1.99	2.841 (18)	156
O15—H15*B*⋯O7	0.89	2.12	2.887 (19)	143
O16—H16*A*⋯O4^iii^	0.82	2.01	2.808 (19)	165
O16—H16*B*⋯O15^iv^	0.85	1.88	2.72 (2)	166
C8—H8⋯O10^v^	0.93	2.53	3.27 (2)	137
C9—H9⋯O12^v^	0.93	2.54	3.425 (19)	158
C11—H11⋯O11^i^	0.93	2.41	3.24 (2)	148
C15—H15⋯O15^vi^	0.93	2.56	3.28 (2)	135

Symmetry codes: (i) 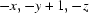; (ii) 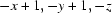; (iii) 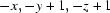; (iv) 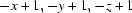; (v) 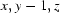; (vi) 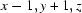.

**Fig. 1.**
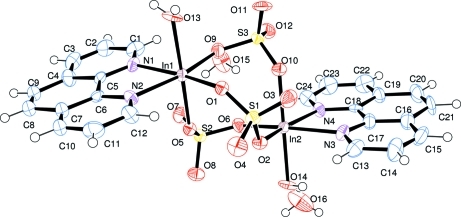
View of the title compound with the atom numbering scheme. Displacement ellipsoids for non-H atoms are drawn at the 50% probability level.

**Fig. 2.**
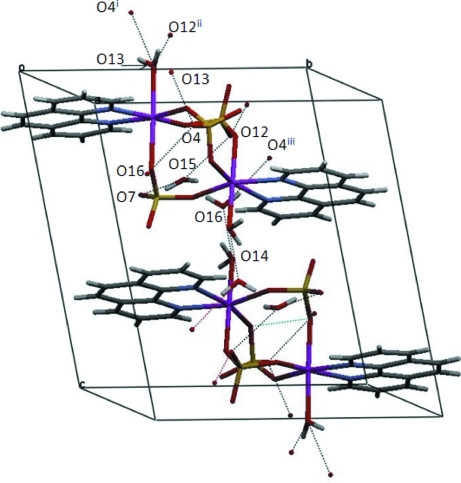
The molecular packing for the title compound. Hydrogen-bonding associations are shown as dotted lines.

## supplementary crystallographic information

### Comment

There are several reports on coordination of Lewis base to indium metal, such as tris(benzimidazol-2-ylmethyl)amine, [InCl_2_(C_24_H_21_N_7_)]Cl (Xiao & Zhan, 2010), 5,5'-dimethyl-2,2'-bipyridine, [InCl_3_(C_12_H_12_N_2_)(CH_4_O)] (Kalateh *et al.*, 2008) and 4,4'-dimethyl-2,2'-bipyridine, [InCl_3_(C_12_H_12_N_2_)(C_2_H_6_OS)] (Ahmadi *et al.*, 2008). The sulfonato ligand exhibits a bridging mode in a In^(III)^ compound (Ramezanipour *et al.*, 2005).

In the structure, the In^III^ atom is six-coordinated within a slightly distorted octahedral coordination geometry defined by 1,10-phenanthroline-N atoms, three sulfate-O atoms and aqua molecule as shown in Fig. 1 and Table 1). In the crystal structure, there are several O—H···O and C—H···O hydrogen bonds (Table 2 and Fig. 2). The supra-molecular structure is consolidated by π-π stacking between nearly parallel phen rings systems [dihedral angle 4.2 (4)°], the centroids distance between benzene rings *Cg*7 (C4—C9)···*Cg*8 (C16—C21) of adjacent phen ligands is 3.528 (9) Å [symmetry code: *x*, -1+y, *z*].

### Experimental

The reaction of benzoic acid (0.176 g, 1.0 mmol) and sodium hydroxide (0.04 g, 1.0 mmol) in methanol/water (1:1, 10 ml) at room temperature for one hour produced a colorless solutions, to which 1,10-phenanthroline (0.218 g, 1 mmol) and In_2_(SO_4_)_3_.6H_2_O (91.0 mg, 0.50 mmol) in water (5 ml) was added. The resulting solution was stirred for one hour at 323 K and the precipitate was filtered. Colorless crystals were obtained by slow evaporation of the filtrate for more than five days.

### Refinement

The water H atoms were placed in chemical sensible positions and refined in riding mode with U_iso_(H) = 1.5U_eq_(O). The remaining H atoms were positioned geometrically and refined using a riding model with C—H = 0.93 Å (aromatic) and U_iso_(H) = 1.2U_eq_(C). The precise of the structure is low.

### Crystal data

**Table:**

[In_2_(SO_4_)_3_(C_12_H_8_N_2_)_2_(H_2_O)_2_]·2H_2_O	*Z* = 2
*M**_r_* = 950.32	*F*(000) = 940
Triclinic, *P*1	*D*_x_ = 2.097 Mg m^−^^3^
Hall symbol: -P 1	Mo *K*α radiation, λ = 0.71073 Å
*a* = 10.5909 (2) Å	Cell parameters from 10717 reflections
*b* = 12.0354 (2) Å	θ = 2.0–25.0°
*c* = 13.3593 (3) Å	µ = 1.83 mm^−^^1^
α = 79.904 (1)°	*T* = 295 K
β = 79.606 (1)°	Prism, colorless
γ = 64.727 (1)°	0.18 × 0.12 × 0.04 mm
*V* = 1505.32 (5) Å^3^	

### Data collection

**Table:**

Bruker SMART CCD area-detector diffractometer	5277 independent reflections
Radiation source: fine-focus sealed tube	4377 reflections with *I* > 2σ(*I*)
graphite	*R*_int_ = 0.079
Detector resolution: 9 pixels mm^-1^	θ_max_ = 25.0°, θ_min_ = 2.3°
ω scan	*h* = −12→12
Absorption correction: multi-scan (*SADABS*; Bruker, 2000)	*k* = −13→14
*T*_min_ = 0.789, *T*_max_ = 0.850	*l* = −15→15
14402 measured reflections	

### Refinement

**Table:**

Refinement on *F*^2^	Primary atom site location: structure-invariant direct methods
Least-squares matrix: full	Secondary atom site location: difference Fourier map
*R*[*F*^2^ > 2σ(*F*^2^)] = 0.082	Hydrogen site location: inferred from neighbouring sites
*wR*(*F*^2^) = 0.224	H-atom parameters constrained
*S* = 1.17	*w* = 1/[σ^2^(*F*_o_^2^) + (0.0492*P*)^2^ + 58.7561*P*] where *P* = (*F*_o_^2^ + 2*F*_c_^2^)/3
5277 reflections	(Δ/σ)_max_ = 0.003
406 parameters	Δρ_max_ = 3.41 e Å^−^^3^
0 restraints	Δρ_min_ = −1.32 e Å^−^^3^

### Special details

**Table:**

Geometry. All esds (except the esd in the dihedral angle between two l.s. planes) are estimated using the full covariance matrix. The cell esds are taken into account individually in the estimation of esds in distances, angles and torsion angles; correlations between esds in cell parameters are only used when they are defined by crystal symmetry. An approximate (isotropic) treatment of cell esds is used for estimating esds involving l.s. planes.
Refinement. Refinement on *F*^2^ for ALL reflections except those flagged by the user for potential systematic errors. Weighted *R*-factors *wR* and all goodnesses of fit *S* are based on *F*^2^, conventional *R*-factors *R* are based on *F*, with *F* set to zero for negative *F*^2^. The observed criterion of *F*^2^ > σ(*F*^2^) is used only for calculating -*R*-factor-obs *etc*. and is not relevant to the choice of reflections for refinement. *R*-factors based on *F*^2^ are statistically about twice as large as those based on *F*, and *R*-factors based on ALL data will be even larger.

### Fractional atomic coordinates and isotropic or equivalent isotropic displacement parameters (Å^2^)

**Table:**

	*x*	*y*	*z*	*U*_iso_*/*U*_eq_	
In1	0.23687 (10)	0.37849 (8)	0.12705 (7)	0.0204 (3)	
In2	0.15197 (10)	0.63043 (9)	0.34430 (7)	0.0205 (3)	
S1	−0.0558 (3)	0.6360 (3)	0.1863 (3)	0.0227 (7)	
S2	0.2944 (4)	0.3255 (3)	0.3720 (3)	0.0226 (7)	
S3	0.3709 (3)	0.5796 (3)	0.1245 (3)	0.0225 (7)	
O1	0.0541 (10)	0.5445 (9)	0.1186 (7)	0.030 (2)	
O2	−0.0126 (9)	0.6094 (9)	0.2926 (7)	0.0259 (12)	
O3	−0.0774 (11)	0.7586 (10)	0.1424 (8)	0.037 (3)	
O4	−0.1878 (11)	0.6163 (11)	0.1984 (8)	0.037 (3)	
O5	0.1989 (9)	0.3469 (9)	0.2939 (7)	0.0259 (12)	
O6	0.3012 (10)	0.4480 (9)	0.3727 (8)	0.029 (2)	
O7	0.4358 (11)	0.2357 (10)	0.3495 (8)	0.035 (2)	
O8	0.2261 (10)	0.2921 (10)	0.4705 (8)	0.032 (2)	
O9	0.3816 (10)	0.4486 (9)	0.1378 (8)	0.031 (2)	
O10	0.2435 (9)	0.6503 (9)	0.1889 (7)	0.0259 (12)	
O11	0.3554 (11)	0.6339 (10)	0.0190 (8)	0.041 (3)	
O12	0.4971 (11)	0.5716 (10)	0.1598 (8)	0.035 (2)	
O13	0.2881 (10)	0.4015 (10)	−0.0407 (7)	0.0319 (17)	
H13A	0.2306	0.4310	−0.0813	0.048*	
H13B	0.3722	0.3720	−0.0684	0.048*	
O14	0.0542 (10)	0.6127 (10)	0.4992 (7)	0.0319 (17)	
H14A	−0.0315	0.6365	0.5110	0.048*	
H14B	0.0906	0.6270	0.5466	0.048*	
N1	0.4033 (12)	0.1854 (10)	0.1249 (9)	0.026 (3)	
N2	0.1239 (12)	0.2648 (11)	0.1099 (9)	0.024 (2)	
N3	0.0258 (13)	0.8347 (11)	0.3484 (9)	0.029 (3)	
N4	0.2974 (13)	0.7026 (11)	0.3855 (9)	0.027 (3)	
C1	0.5403 (15)	0.1495 (15)	0.1316 (12)	0.036 (4)	
H1	0.5755	0.2084	0.1308	0.043*	
C2	0.6308 (16)	0.0235 (15)	0.1399 (13)	0.039 (4)	
H2	0.7252	−0.0002	0.1458	0.047*	
C3	0.5828 (18)	−0.0640 (14)	0.1396 (12)	0.038 (4)	
H3	0.6439	−0.1472	0.1440	0.046*	
C4	0.4372 (17)	−0.0271 (13)	0.1323 (12)	0.035 (4)	
C5	0.3541 (15)	0.0995 (13)	0.1217 (10)	0.025 (3)	
C6	0.2044 (13)	0.1400 (13)	0.1147 (10)	0.023 (3)	
C7	0.1516 (18)	0.0558 (15)	0.1097 (11)	0.034 (2)	
C8	0.241 (2)	−0.0743 (14)	0.1168 (13)	0.043 (3)	
H8	0.2051	−0.1314	0.1128	0.051*	
C9	0.377 (2)	−0.1128 (15)	0.1294 (13)	0.043 (3)	
H9	0.4334	−0.1971	0.1365	0.051*	
C10	0.0089 (18)	0.1022 (15)	0.0970 (11)	0.034 (2)	
H10	−0.0299	0.0478	0.0907	0.041*	
C11	−0.0732 (17)	0.2247 (17)	0.0936 (13)	0.043 (4)	
H11	−0.1684	0.2547	0.0869	0.051*	
C12	−0.0121 (15)	0.3057 (14)	0.1005 (12)	0.033 (3)	
H12	−0.0681	0.3900	0.0985	0.040*	
C13	−0.1113 (17)	0.8982 (16)	0.3400 (12)	0.038 (4)	
H13	−0.1635	0.8547	0.3339	0.046*	
C14	−0.181 (2)	1.0270 (15)	0.3400 (14)	0.049 (5)	
H14	−0.2748	1.0686	0.3295	0.059*	
C15	−0.106 (2)	1.0893 (16)	0.3558 (12)	0.045 (4)	
H15	−0.1511	1.1744	0.3579	0.054*	
C16	0.0386 (19)	1.0274 (15)	0.3690 (12)	0.040 (4)	
C17	0.0940 (16)	0.9010 (14)	0.3676 (11)	0.030 (3)	
C18	0.2413 (15)	0.8307 (13)	0.3847 (10)	0.026 (3)	
C19	0.3237 (18)	0.8895 (14)	0.3983 (12)	0.035 (4)	
C20	0.260 (2)	1.0237 (17)	0.3947 (13)	0.049 (5)	
H20	0.3137	1.0650	0.4022	0.058*	
C21	0.126 (2)	1.0879 (15)	0.3809 (14)	0.048 (5)	
H21	0.0869	1.1736	0.3789	0.057*	
C22	0.4654 (19)	0.8164 (19)	0.4137 (14)	0.048 (3)	
H22	0.5225	0.8539	0.4233	0.057*	
C23	0.5219 (19)	0.6850 (19)	0.4148 (14)	0.048 (3)	
H23	0.6148	0.6349	0.4253	0.057*	
C24	0.4313 (16)	0.6369 (16)	0.3995 (12)	0.035 (4)	
H24	0.4665	0.5517	0.3989	0.042*	
O15	0.6347 (12)	0.3469 (12)	0.2823 (12)	0.059 (4)	
H15A	0.5690	0.4170	0.2546	0.089*	
H15B	0.6100	0.2850	0.3113	0.089*	
O16	0.1901 (16)	0.5776 (15)	0.6508 (10)	0.069 (5)	
H16A	0.2024	0.5127	0.6878	0.104*	
H16B	0.2324	0.6122	0.6739	0.104*	

### Atomic displacement parameters (Å^2^)

**Table:**

	*U*^11^	*U*^22^	*U*^33^	*U*^12^	*U*^13^	*U*^23^
In1	0.0203 (5)	0.0179 (5)	0.0237 (5)	−0.0076 (4)	−0.0032 (4)	−0.0041 (4)
In2	0.0198 (5)	0.0197 (5)	0.0224 (5)	−0.0078 (4)	−0.0016 (4)	−0.0048 (4)
S1	0.0180 (16)	0.0237 (17)	0.0250 (17)	−0.0057 (13)	−0.0037 (13)	−0.0053 (13)
S2	0.0238 (17)	0.0209 (17)	0.0225 (17)	−0.0089 (14)	−0.0027 (13)	−0.0015 (13)
S3	0.0217 (17)	0.0234 (17)	0.0238 (17)	−0.0114 (14)	−0.0011 (13)	−0.0022 (13)
O1	0.024 (5)	0.028 (5)	0.028 (5)	0.002 (4)	−0.006 (4)	−0.011 (4)
O2	0.020 (3)	0.034 (3)	0.023 (3)	−0.015 (2)	−0.004 (2)	0.008 (2)
O3	0.038 (6)	0.026 (6)	0.034 (6)	−0.002 (5)	−0.003 (5)	−0.003 (5)
O4	0.033 (6)	0.049 (7)	0.036 (6)	−0.020 (5)	−0.016 (5)	−0.003 (5)
O5	0.020 (3)	0.034 (3)	0.023 (3)	−0.015 (2)	−0.004 (2)	0.008 (2)
O6	0.031 (5)	0.023 (5)	0.031 (5)	−0.010 (4)	−0.004 (4)	0.002 (4)
O7	0.027 (5)	0.028 (6)	0.041 (6)	−0.004 (5)	−0.004 (5)	−0.002 (5)
O8	0.025 (5)	0.038 (6)	0.030 (5)	−0.012 (5)	−0.003 (4)	0.002 (5)
O9	0.030 (6)	0.027 (5)	0.037 (6)	−0.013 (5)	0.000 (4)	−0.008 (4)
O10	0.020 (3)	0.034 (3)	0.023 (3)	−0.015 (2)	−0.004 (2)	0.008 (2)
O11	0.039 (6)	0.038 (6)	0.023 (5)	−0.006 (5)	0.014 (5)	0.008 (5)
O12	0.027 (5)	0.036 (6)	0.046 (6)	−0.018 (5)	0.002 (5)	−0.006 (5)
O13	0.023 (4)	0.052 (5)	0.022 (4)	−0.025 (4)	0.003 (3)	0.009 (3)
O14	0.023 (4)	0.052 (5)	0.022 (4)	−0.025 (4)	0.003 (3)	0.009 (3)
N1	0.030 (7)	0.020 (6)	0.024 (6)	−0.005 (5)	−0.008 (5)	−0.004 (5)
N2	0.023 (6)	0.026 (6)	0.026 (6)	−0.013 (5)	−0.006 (5)	0.002 (5)
N3	0.030 (7)	0.027 (7)	0.029 (7)	−0.009 (5)	−0.004 (5)	−0.011 (5)
N4	0.036 (7)	0.026 (6)	0.024 (6)	−0.018 (6)	−0.010 (5)	−0.001 (5)
C1	0.017 (7)	0.039 (9)	0.040 (9)	0.001 (6)	−0.006 (6)	−0.005 (7)
C2	0.020 (7)	0.035 (9)	0.046 (10)	0.004 (7)	−0.005 (7)	−0.002 (7)
C3	0.044 (10)	0.023 (8)	0.034 (9)	0.004 (7)	−0.015 (7)	0.000 (6)
C4	0.039 (9)	0.018 (7)	0.042 (9)	−0.006 (7)	−0.007 (7)	0.004 (6)
C5	0.035 (8)	0.023 (7)	0.016 (6)	−0.011 (6)	0.001 (6)	−0.007 (5)
C6	0.015 (6)	0.026 (7)	0.025 (7)	−0.007 (6)	0.005 (5)	−0.010 (6)
C7	0.051 (7)	0.041 (6)	0.026 (5)	−0.033 (6)	0.001 (5)	−0.008 (5)
C8	0.059 (7)	0.022 (6)	0.044 (7)	−0.018 (6)	0.010 (6)	−0.007 (5)
C9	0.059 (7)	0.022 (6)	0.044 (7)	−0.018 (6)	0.010 (6)	−0.007 (5)
C10	0.051 (7)	0.041 (6)	0.026 (5)	−0.033 (6)	0.001 (5)	−0.008 (5)
C11	0.027 (8)	0.056 (11)	0.047 (10)	−0.019 (8)	0.000 (7)	−0.009 (8)
C12	0.022 (7)	0.027 (8)	0.047 (9)	−0.008 (6)	−0.007 (7)	0.002 (7)
C13	0.034 (9)	0.039 (9)	0.032 (8)	−0.005 (7)	−0.006 (7)	−0.008 (7)
C14	0.053 (11)	0.026 (9)	0.050 (11)	0.006 (8)	−0.022 (9)	0.000 (8)
C15	0.057 (11)	0.031 (9)	0.032 (9)	−0.002 (8)	0.000 (8)	−0.013 (7)
C16	0.057 (11)	0.028 (8)	0.032 (9)	−0.014 (8)	−0.007 (8)	−0.003 (7)
C17	0.034 (8)	0.024 (7)	0.031 (8)	−0.010 (6)	−0.005 (6)	−0.004 (6)
C18	0.030 (8)	0.030 (8)	0.015 (6)	−0.014 (6)	0.009 (5)	−0.002 (6)
C19	0.048 (10)	0.028 (8)	0.035 (8)	−0.022 (7)	−0.004 (7)	−0.004 (7)
C20	0.084 (15)	0.038 (10)	0.040 (10)	−0.039 (11)	−0.008 (9)	−0.006 (8)
C21	0.075 (14)	0.019 (8)	0.053 (11)	−0.025 (9)	0.000 (10)	−0.005 (7)
C22	0.042 (7)	0.066 (8)	0.048 (7)	−0.029 (7)	−0.012 (6)	−0.013 (7)
C23	0.042 (7)	0.066 (8)	0.048 (7)	−0.029 (7)	−0.012 (6)	−0.013 (7)
C24	0.030 (8)	0.040 (9)	0.037 (9)	−0.015 (7)	−0.013 (7)	0.003 (7)
O15	0.028 (6)	0.042 (7)	0.107 (12)	−0.014 (6)	−0.023 (7)	0.009 (7)
O16	0.086 (11)	0.102 (12)	0.050 (8)	−0.071 (10)	−0.044 (8)	0.038 (8)

### Geometric parameters (Å, °)

**Table:**

In1—N1	2.240 (11)	C3—C4	1.43 (2)
In1—N2	2.228 (11)	C3—H3	0.9300
In1—O1	2.109 (9)	C4—C5	1.39 (2)
In1—O5	2.187 (9)	C4—C9	1.43 (2)
In1—O9	2.075 (10)	C5—C6	1.462 (19)
In1—O13	2.206 (9)	C6—C7	1.37 (2)
In2—N3	2.247 (12)	C7—C10	1.40 (2)
In2—N4	2.246 (11)	C7—C8	1.44 (2)
In2—O2	2.109 (9)	C8—C9	1.34 (3)
In2—O6	2.103 (10)	C8—H8	0.9300
In2—O10	2.146 (9)	C9—H9	0.9300
In2—O14	2.159 (9)	C10—C11	1.35 (2)
S1—O3	1.425 (11)	C10—H10	0.9300
S1—O4	1.491 (11)	C11—C12	1.40 (2)
S1—O1	1.504 (10)	C11—H11	0.9300
S1—O2	1.509 (10)	C12—H12	0.9300
S2—O7	1.440 (10)	C13—C14	1.40 (2)
S2—O8	1.461 (10)	C13—H13	0.9300
S2—O5	1.497 (10)	C14—C15	1.36 (3)
S2—O6	1.509 (10)	C14—H14	0.9300
S3—O11	1.451 (10)	C15—C16	1.42 (3)
S3—O12	1.456 (11)	C15—H15	0.9300
S3—O10	1.464 (10)	C16—C17	1.38 (2)
S3—O9	1.512 (10)	C16—C21	1.44 (2)
O13—H13A	0.8112	C17—C18	1.46 (2)
O13—H13B	0.8450	C18—C19	1.39 (2)
O14—H14A	0.8198	C19—C22	1.41 (2)
O14—H14B	0.8726	C19—C20	1.46 (2)
N1—C1	1.341 (19)	C20—C21	1.33 (3)
N1—C5	1.351 (18)	C20—H20	0.9300
N2—C12	1.331 (18)	C21—H21	0.9300
N2—C6	1.371 (18)	C22—C23	1.43 (3)
N3—C13	1.34 (2)	C22—H22	0.9300
N3—C17	1.362 (19)	C23—C24	1.37 (2)
N4—C24	1.326 (19)	C23—H23	0.9300
N4—C18	1.394 (19)	C24—H24	0.9300
C1—C2	1.40 (2)	O15—H15A	0.9033
C1—H1	0.9300	O15—H15B	0.8951
C2—C3	1.35 (2)	O16—H16A	0.8201
C2—H2	0.9300	O16—H16B	0.8501
			
O9—In1—O1	99.7 (4)	N1—C1—H1	119.8
O9—In1—O5	91.1 (4)	C2—C1—H1	119.8
O1—In1—O5	93.0 (4)	C3—C2—C1	121.0 (15)
O9—In1—O13	87.6 (4)	C3—C2—H2	119.5
O1—In1—O13	90.4 (4)	C1—C2—H2	119.5
O5—In1—O13	176.5 (4)	C2—C3—C4	119.2 (14)
O9—In1—N2	167.3 (4)	C2—C3—H3	120.4
O1—In1—N2	92.8 (4)	C4—C3—H3	120.4
O5—In1—N2	90.8 (4)	C5—C4—C3	116.8 (14)
O13—In1—N2	89.8 (4)	C5—C4—C9	119.8 (15)
O9—In1—N1	92.2 (4)	C3—C4—C9	123.3 (14)
O1—In1—N1	167.8 (4)	N1—C5—C4	123.0 (14)
O5—In1—N1	89.7 (4)	N1—C5—C6	118.8 (12)
O13—In1—N1	87.2 (4)	C4—C5—C6	118.0 (13)
N2—In1—N1	75.3 (4)	C7—C6—N2	123.0 (13)
O6—In2—O2	103.9 (4)	C7—C6—C5	120.3 (13)
O6—In2—O10	93.0 (4)	N2—C6—C5	116.6 (12)
O2—In2—O10	90.0 (3)	C6—C7—C10	117.1 (15)
O6—In2—O14	88.1 (4)	C6—C7—C8	120.3 (15)
O2—In2—O14	88.6 (4)	C10—C7—C8	122.6 (14)
O10—In2—O14	178.4 (4)	C9—C8—C7	119.7 (16)
O6—In2—N4	90.1 (4)	C9—C8—H8	120.2
O2—In2—N4	165.7 (4)	C7—C8—H8	120.2
O10—In2—N4	86.4 (4)	C8—C9—C4	121.6 (15)
O14—In2—N4	94.7 (4)	C8—C9—H9	119.2
O6—In2—N3	162.6 (4)	C4—C9—H9	119.2
O2—In2—N3	92.0 (4)	C11—C10—C7	121.1 (14)
O10—In2—N3	93.8 (4)	C11—C10—H10	119.5
O14—In2—N3	85.4 (4)	C7—C10—H10	119.5
N4—In2—N3	74.4 (4)	C10—C11—C12	118.7 (15)
O3—S1—O4	111.4 (7)	C10—C11—H11	120.6
O3—S1—O1	109.8 (6)	C12—C11—H11	120.6
O4—S1—O1	108.3 (6)	N2—C12—C11	121.7 (15)
O3—S1—O2	111.8 (6)	N2—C12—H12	119.2
O4—S1—O2	105.6 (6)	C11—C12—H12	119.2
O1—S1—O2	109.9 (6)	N3—C13—C14	123.5 (17)
O7—S2—O8	112.4 (6)	N3—C13—H13	118.2
O7—S2—O5	114.4 (6)	C14—C13—H13	118.2
O8—S2—O5	106.6 (6)	C15—C14—C13	118.1 (17)
O7—S2—O6	107.7 (6)	C15—C14—H14	121.0
O8—S2—O6	109.7 (6)	C13—C14—H14	121.0
O5—S2—O6	105.9 (6)	C14—C15—C16	121.5 (16)
O11—S3—O12	113.5 (7)	C14—C15—H15	119.3
O11—S3—O10	108.3 (6)	C16—C15—H15	119.3
O12—S3—O10	111.7 (6)	C17—C16—C15	114.6 (16)
O11—S3—O9	110.9 (7)	C17—C16—C21	120.9 (16)
O12—S3—O9	105.8 (6)	C15—C16—C21	124.5 (16)
O10—S3—O9	106.5 (6)	N3—C17—C16	126.2 (15)
S1—O1—In1	140.6 (6)	N3—C17—C18	116.2 (13)
S1—O2—In2	129.8 (5)	C16—C17—C18	117.6 (14)
S2—O5—In1	128.9 (5)	C19—C18—N4	120.9 (13)
S2—O6—In2	131.2 (6)	C19—C18—C17	121.2 (14)
S3—O9—In1	131.6 (6)	N4—C18—C17	118.0 (13)
S3—O10—In2	136.9 (6)	C18—C19—C22	118.4 (15)
In1—O13—H13A	124.8	C18—C19—C20	118.3 (16)
In1—O13—H13B	121.1	C22—C19—C20	123.3 (16)
H13A—O13—H13B	113.7	C21—C20—C19	120.9 (16)
In2—O14—H14A	120.1	C21—C20—H20	119.5
In2—O14—H14B	116.0	C19—C20—H20	119.5
H14A—O14—H14B	112.8	C20—C21—C16	121.1 (15)
C1—N1—C5	119.4 (13)	C20—C21—H21	119.4
C1—N1—In1	126.4 (11)	C16—C21—H21	119.4
C5—N1—In1	114.1 (9)	C19—C22—C23	120.3 (16)
C12—N2—C6	118.5 (12)	C19—C22—H22	119.8
C12—N2—In1	126.6 (10)	C23—C22—H22	119.8
C6—N2—In1	114.9 (8)	C24—C23—C22	116.3 (17)
C13—N3—C17	115.7 (13)	C24—C23—H23	121.9
C13—N3—In2	127.4 (11)	C22—C23—H23	121.9
C17—N3—In2	116.7 (9)	N4—C24—C23	125.0 (16)
C24—N4—C18	119.1 (13)	N4—C24—H24	117.5
C24—N4—In2	126.0 (10)	C23—C24—H24	117.5
C18—N4—In2	114.5 (9)	H15A—O15—H15B	118.7
N1—C1—C2	120.4 (16)	H16A—O16—H16B	106.6

### Hydrogen-bond geometry (Å, °)

**Table:**

*D*—H···*A*	*D*—H	H···*A*	*D*···*A*	*D*—H···*A*
O13—H13A···O4^i^	0.81	1.94	2.602 (15)	138
O13—H13B···O12^ii^	0.84	1.94	2.637 (16)	139
O14—H14A···O8^iii^	0.82	1.85	2.667 (17)	173
O14—H14B···O16	0.87	1.76	2.565 (19)	152
O15—H15A···O12	0.90	1.99	2.841 (18)	156
O15—H15B···O7	0.89	2.12	2.887 (19)	143
O16—H16A···O4^iii^	0.82	2.01	2.808 (19)	165
O16—H16B···O15^iv^	0.85	1.88	2.72 (2)	166
C8—H8···O10^v^	0.93	2.53	3.27 (2)	137
C9—H9···O12^v^	0.93	2.54	3.425 (19)	158
C11—H11···O11^i^	0.93	2.41	3.24 (2)	148
C15—H15···O15^vi^	0.93	2.56	3.28 (2)	135

Symmetry codes: (i) −*x*, −*y*+1, −*z*; (ii) −*x*+1, −*y*+1, −*z*; (iii) −*x*, −*y*+1, −*z*+1; (iv) −*x*+1, −*y*+1, −*z*+1; (v) *x*, *y*−1, *z*; (vi) *x*−1, *y*+1, *z*.
